# Dependences of microstructure on electromagnetic interference shielding properties of nano-layered Ti_3_AlC_2_ ceramics

**DOI:** 10.1038/s41598-018-26256-0

**Published:** 2018-05-21

**Authors:** Yongqiang Tan, Heng Luo, Xiaosong Zhou, Shuming Peng, Haibin Zhang

**Affiliations:** Innovation Research Team for Advanced Ceramics, Institute of Nuclear Physics and Chemistry, China Academy of Engineering Physics, Mianyang, 621900 China

## Abstract

The microstructure dependent electromagnetic interference (EMI) shielding properties of nano-layered Ti_3_AlC_2_ ceramics were presented in this study by comparing the shielding properties of various Ti_3_AlC_2_ ceramics with distinct microstructures. Results indicate that Ti_3_AlC_2_ ceramics with dense microstructure and coarse grains are more favourable for superior EMI shielding efficiency. High EMI shielding effectiveness over 40 dB at the whole Ku-band frequency range was achieved in Ti_3_AlC_2_ ceramics by microstructure optimization, and the high shielding effectiveness were well maintained up to 600 °C. A further investigation reveals that only the absorption loss displays variations upon modifying microstructure by allowing more extensive multiple reflections in coarse layered grains. Moreover, the absorption loss of Ti_3_AlC_2_ was found to be much higher than those of highly conductive TiC ceramics without layered structure. These results demonstrate that nano-layered MAX phase ceramics are promising candidates of high-temperature structural EMI shielding materials and provide insightful suggestions for achieving high EMI shielding efficiency in other ceramic-based shielding materials.

## Introduction

Electromagnetic interference (EMI), which could cause detrimental effects on the performance of electronic devices, has drawn growing attentions with the repaid development of highly sensitive circuits^1,2^. Ceramic-based EMI shielding materials have been identified as novel electromagnetic shielding options in the past few years owning to their promising applications as light-weight structural EMI shielding components in areas of aircraft and aerospace^3–6^. Compared to traditional metal- and carbon-based EMI shielding materials, ceramic composites exhibit the great advantage of being mechanically stiff and thermo-stable, which could guarantee their functionality at harsh environments^6^. Besides, the relatively high permittivity and dielectric loss of ceramics are favourable for the attenuation of EMI waves^4,5,7–9^. Nevertheless, the commonly inferior electrical conductivity becomes a major obstacle to obtain high EMI shielding effectiveness (SE) in ceramics. Although the electrical conductivity and EMI SE of ceramics could be both significantly enhanced by incorporating highly conductive carbon materials such as carbon nanotubes (CNTs)^4,10^, carbon fibers (C_f_)^6,11^, graphene nanoplatelets (GNPs)^5,7,8,12^, and pyrolytic carbon^13,14^, the incorporation of those carbon nanostructures in ceramic composites is hindered by the difficulty of homogenous dispersion and their poor oxidation resistance at high temperatures. Accordingly, it is of great interest to explore intrinsically conductive ceramics as effective high-temperature EMI shielding materials.

MAX phase ceramics which represent a class of layered ternary transition-metal carbides and nitrides with a general formula of M_n+1_AX_n_ (wherein M is an early transition metal, A is an A-group element, X is either C or N, and n varies from 1 to 3)^15^ are ideal candidates of high-temperature structural EMI shielding materials owning to their satisfactory mechanical properties, intrinsically superior electrical conductivity and the nano-layered structure^15,16^. Ti_3_AlC_2_ and Ti_3_SiC_2_ are two most studied MAX phases and both have been reported to exhibit high EMI SE^17,18^. Preliminary investigation demonstrates that the nano-layered structure of MAX phases made a considerable contribution to the total shielding effectiveness^17^.

As was well known, the physical properties of composites usually show remarkable variations upon the change of their microstructures such as porosity, average grain size and interfaces^2,19–22^. The physical properties of ceramics could be possibly enhanced by a large scale through the optimization of microstructures. Especially for highly conductive nano-layered structures, the grain size, aspect ratios and the alignment are supposed to have a decisive influence on the EMI shielding properties^20^. For example, significantly enhanced shielding efficiency was recently reported in highly aligned graphene/polymer nanocomposites^20^. However, no studies on the dependences of microstructures on EMI shielding properties of ceramics have been reported to our best knowledge. Accordingly, it is of great interest to study the effects of microstructure on the EMI shielding properties not only to obtain excellent high-temperature EMI shielding properties in MAX phase ceramics but also to unfold the origin of high EMI SE in ceramics with unique microstructure. Compared to Ti_3_SiC_2_, Ti_3_AlC_2_ exhibits much better oxidation resistance at high temperatures^23^ and is therefore more suitable for high-temperature applications. Accordingly, Ti_3_AlC_2_ was chosen as study object, and four typical types of nano-layered Ti_3_AlC_2_ ceramics with different microstructures were fabricated by pressureless and hot-press sintering as well as their combination. As a comparison, the EMI shielding properties of highly conductive TiC ceramics without nano-layered structure were also examined. The influences of microstructure on the high-temperature EMI shielding properties were investigated systematically, and the mechanisms for high EMI SE in Ti_3_AlC_2_ ceramics were presented.

## Results and Discussion

**Figure 1:**
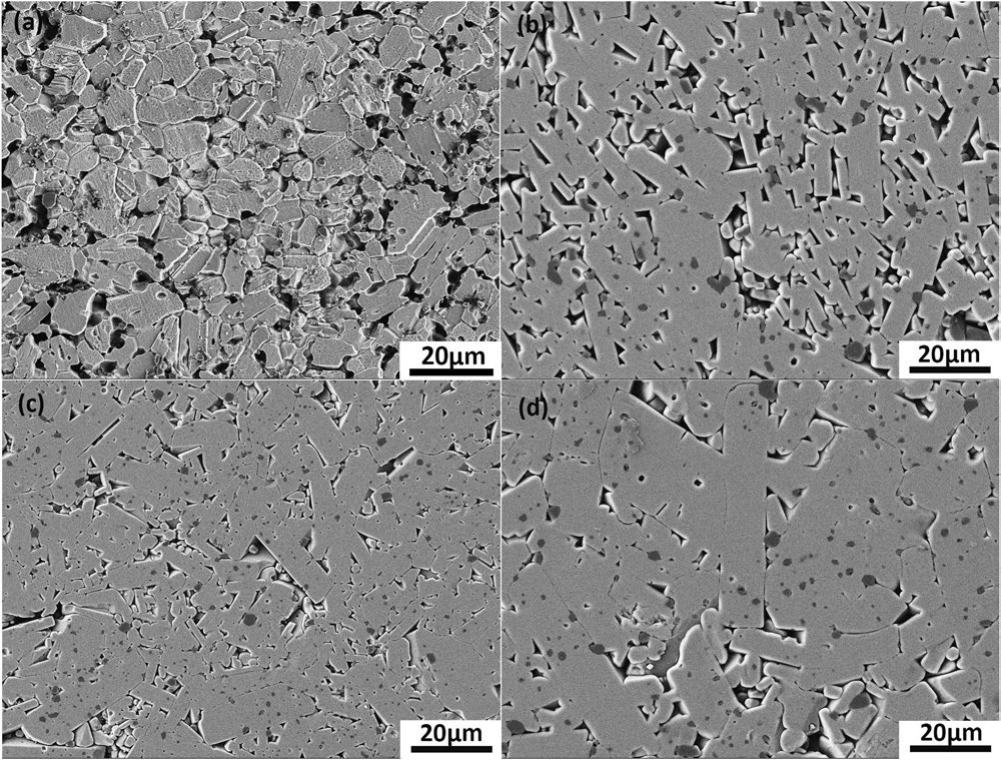
SEM images of polished surfaces of various Ti_3_AlC_2_ ceramics with distinct microstructures. (**a**) TAC-A; (**b**) TAC-B; (**c**) TAC-C; (**d**) TAC-D. All Ti_3_AlC_2_ ceramics show typical layered grains. The pressureless sintered sample shows certain degree of pores. With the increase of hot-pressing temperature, the microstructure becomes denser and shows significant grain growth.

Figure 1 exhibits the SEM images of polished and etched surfaces of the various Ti_3_AlC_2_ ceramics prepared by different methods (given in Table 1). Obviously, all Ti_3_AlC_2_ ceramics exhibit distinct microstructures. The pressureless-sintered sample shows certain amounts of pores, which is consistent with its relatively lower relative density around 91% as indicated in Table 1. Besides, the aspect ratio of pressureless-sintered Ti_3_AlC_2_ grains is relatively low due to the absence of pressure during sintering. All hot-pressed Ti_3_AlC_2_ ceramics exhibit dense microstructures and layered grains with higher aspect ratios. The distributions of grain level dimension for different Ti_3_AlC_2_ ceramics were statistically calculated and exhibited in Fig. S1. The majority of grains for pressureless-sintered Ti_3_AlC_2_ ceramic (TAC-A) show level dimensions in the range of 8–14 µm. The average level dimension of hot-pressed samples increases significantly with increasing sintering temperature and duration, which is consistent with previous study^24^. Both the Ti_3_AlC_2_ ceramics sintered at 1350 °C for 15 min (TAC-B) and 1400 °C for 2 h (TAC-C) show broad level dimension distributions. The TAC-B shows a level dimension peak around 10 µm while the distribution peak of TAC-C locates at a higher size range around 20 µm. A followed thermal process at 1500 °C for 10 h further promotes the grain growth with a dominant percentage of grains with level dimension larger than 25 µm and a considerable percentage of grains with level dimension larger than 40 µm. All the Ti_3_AlC_2_ ceramics exhibit the same single-phase crystal structure without detectable second phases, which can be evidenced by the XRD patterns shown in Fig. S2 in the supporting information.

**Table 1:** Comparison of relative density and electrical conductivity of various Ti_3_AlC_2_ and TiC ceramics.

Sample	Preparation Conditions	Relative Density	Electrical Conductivity
TAC-A	Pressureless sintering (1500 °C, 2 h)	91.2%	2.4*10^6^ S/m
TAC-B	Hot-pressing (1350 °C, 15 min)	95.6%	2.9*10^6^ S/m
TAC-C	Hot-pressing (1400 °C, 2 h)	98.5%	3.0*10^6^ S/m
TAC-D	Hot-pressing (1400 °C,2 h) + Pressureless sintering (1500 °C, 10 h)	98.2%	3.2*10^6^ S/m
TiC-A	Hot-pressing (1800 °C, 15 min)	98.6%	4.2*10^6^ S/m
TiC-B	Hot-pressing (1950 °C, 2 h)	98.9%	4.7*10^6^ S/m

The total SE (SE_T_) of EMI shielding materials can be characterized as follows^25^:

1 $${{\rm{S}}{\rm{E}}}_{{\rm{T}}}=10\,{\rm{l}}{\rm{o}}{\rm{g}}({{\rm{P}}}_{{\rm{i}}}/{{\rm{P}}}_{{\rm{t}}})={{\rm{S}}{\rm{E}}}_{{\rm{R}}}+{{\rm{S}}{\rm{E}}}_{{\rm{A}}}+{{\rm{S}}{\rm{E}}}_{{\rm{M}}}$$

in which SE_T_, SE_A_ SE_R_ and SE_M_ denote the total SE, SE due to reflection, absorption loss and multiple reflections, respectively. SE_T_, SE_R_ and SE_A_ were calculated from the *S*-parameters as follows^26^,

2 $${{\rm{S}}{\rm{E}}}_{{\rm{T}}}=-\,10\,{\rm{l}}{\rm{o}}{\rm{g}}\,{|{{\rm{S}}}_{12}|}^{2}$$

3 $${{\rm{S}}{\rm{E}}}_{{\rm{R}}}=-\,10\,{\rm{l}}{\rm{o}}{\rm{g}}(1-{|{{\rm{S}}}_{11}|}^{2})$$

4 $${{\rm{S}}{\rm{E}}}_{{\rm{A}}}=-\,10\,{\rm{l}}{\rm{o}}{\rm{g}}(\frac{{|{{\rm{S}}}_{21}|}^{2}}{1-{|{{\rm{S}}}_{11}|}^{2}})$$

**Figure 2:**
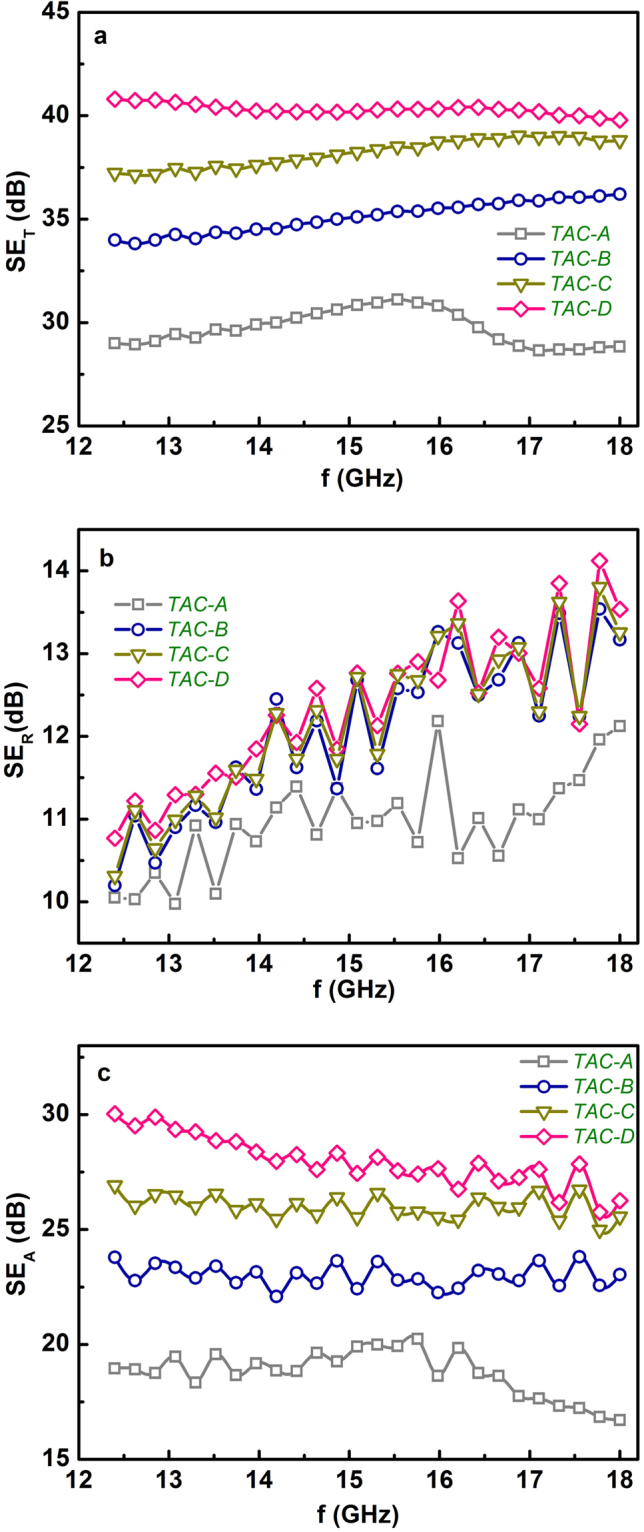
The frequency dependences of (**a**) the total shielding effectiveness SE_T_, (**b**) reflection loss SE_R_ and (**c**) absorption loss SE_A_ of various Ti_3_AlC_2_ ceramics with distinct microstructures measured at room-temperature. Ti_3_AlC_2_ ceramics with various microstructures exhibit different EMI shielding capability and those differences mainly arose from the distinct absorption loss.

Figure 2a–c compare the frequency dependences of room-temperature SE_T_, SE_R_ and SE_A_ respectively of Ti_3_AlC_2_ ceramics with different microstructures. The SE_T_ exhibits notable differences among various Ti_3_AlC_2_ ceramics. The pressureless sintered Ti_3_AlC_2_ ceramic exhibits the lowest SE_T_ around 30 dB, which is comparable to those of most ceramic-based composites^5,11,27^. The hot-pressed Ti_3_AlC_2_ ceramics exhibit remarkably enhanced SE_T_ compared to pressureless sintered sample. Further, the room-temperature SE_T_ of hot-pressed samples increases monotonically with the increase of grain size. TAC-D ceramic with the largest grain size shows the highest SE_T_ over 40 dB at the whole Ku-band, which means over 99.99% of the incident radiation could be effectively blocked with only 0.01% transmission. The room-temperature EMI SE_T_ of TAC-D ceramic is higher than most ceramic-based EMI shielding composites containing conductive particles or carbon nano-fillers^3–6,8,27^, while the preparation of Ti_3_AlC_2_ ceramics is much less complicated. Moreover, the high SE_T_ of TAC-D ceramic is almost independent of the frequency. In Equation (1), the SE_A_ can be regarded as the energy dissipation of the electromagnetic microwave in the absorber and therefore the multiple reflection SE_M_ in single phase Ti_3_AlC_2_ ceramics actually is included in absorption because the re-reflected waves could get absorbed or dissipated within the material^1^. Accordingly, the SE_T_ could be expressed as,

5 $${{\rm{S}}{\rm{E}}}_{{\rm{T}}}={{\rm{S}}{\rm{E}}}_{{\rm{R}}}+{{\rm{S}}{\rm{E}}}_{{\rm{A}}}$$

**Figure 3:**
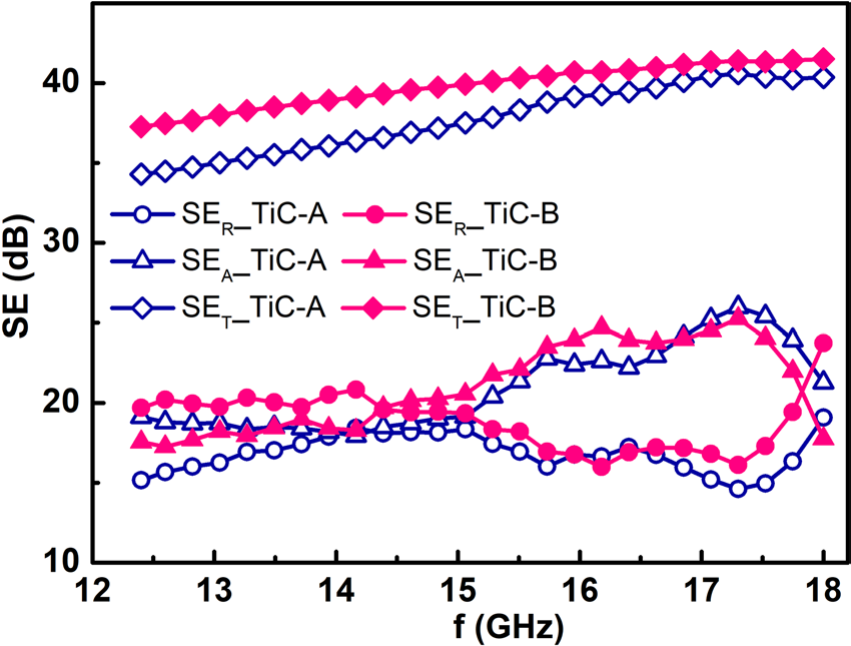
Comparison of the shielding effectiveness of TiC ceramics with different grain size. The shielding effectiveness was measured at room temperature. The shielding capability of TiC ceramics shows much weaker grain size dependence compared to Ti_3_AlC_2_ ceramics.

In order to have a deeper understanding on the origin of microstructure-dependent EMI shielding properties in Ti_3_AlC_2_ ceramics, the dependences of microstructure on the reflection loss SE_R_ and absorption loss SE_A_ of Ti_3_AlC_2_ ceramics were investigated and the results were exhibited in Fig. 3b and c respectively. It can be seen that the SE_R_ of Ti_3_AlC_2_ ceramics is almost independent of their microstructure. All Ti_3_AlC_2_ ceramics with various microstructures show similar SE_R_ values between 10 and 14 dB, especially, the SE_R_ of all the hot-pressed ceramics exhibit almost the same values at the whole Ku-band frequency range. The reflection loss of EMI shielding materials arises mainly from the impedance mismatch between the sample and the free space^25^. The extremely high electrical conductivity (~10^6^ S/m) of Ti_3_AlC_2_ ceramics could lead to a huge impedance mismatch and therefore give rise to a high reflection loss. The pressureless sintered Ti_3_AlC_2_ ceramics exhibit inferior electrical conductivity due to high porosity (Table 1), accordingly, its SE_R_ is relatively lower. While for dense Ti_3_AlC_2_ ceramics, the influence of grain size on the conductivity is negligible and therefore the SE_R_ becomes almost independent of the microstructure. All Ti_3_AlC_2_ ceramics exhibit high absorption loss SE_A_ (Fig. 2c) which is much larger than SE_R_, indicating that Ti_3_AlC_2_ ceramics are typically absorption-dominant EMI shielding materials. Specially, high SE_A_ of 30 dB which is almost 3 times larger than SE_R_ was obtained for TAC-D ceramic at 12.4 GHz. Being different from SE_R_, the absorption loss shows obvious grain size dependence and becomes the major contribution to the variation of SE_T_ among Ti_3_AlC_2_ ceramics with different microstructures, as demonstrated in Fig. 2c. Hot-pressed Ti_3_AlC_2_ ceramics exhibit higher SE_A_ than pressureless sintered sample. For hot-pressed Ti_3_AlC_2_ ceramics, the SE_A_ increases gradually with increasing grain size. Therefore, it can be concluded that dense and coarse-grained microstructure with higher aspect ratios is more favorable for high EMI attenuation and the total EMI shielding in nano-layered Ti_3_AlC_2_ ceramics.

In order to further validate the significant contribution of the nano-layered structure to the absorption loss, the EMI shielding properties of highly conductive TiC ceramics without nano-layered structures were characterized and compared with those of Ti_3_AlC_2_ ceramics. Figure S3 shows the fracture surfaces of TiC ceramics sintered at different temperatures. Both TiC ceramics exhibit different microstructures from Ti_3_AlC_2_ ceramics without layered structures. It is apparent that the grain size of TiC-B is much larger than that of TiC-A. The frequency dependences of the EMI SE for different TiC ceramics were shown in Fig. 3. It is interesting to notice that, being different to that of Ti_3_AlC_2_ ceramics, the change of grain size did not bring significant variation of SE_T_ for TiC ceramics. Especially, the SE_A_ for fine- and coarse-grained TiC ceramics are almost the same. Compared to Ti_3_AlC_2_ ceramics, the TiC ceramics shows much lower SE_A_ while higher SE_R_. The microstructure-independent SE_A_ of TiC ceramics varies in the range of 18–25 dB at the whole frequency range. The SE_R_ has almost equal contribution to the total shielding for both TiC ceramics. The different shielding properties between Ti_3_AlC_2_ and TiC further confirms that the nano-layered structure is responsible for the superior and microstructure-dependent SE_A_ in Ti_3_AlC_2_ ceramics. The reflection loss of Ti_3_AlC_2_ ceramics is considerably lower than that of TiC ceramics although both ceramics exhibit similar electrical conductivity as demonstrated in Table 1.

**Figure 4:**
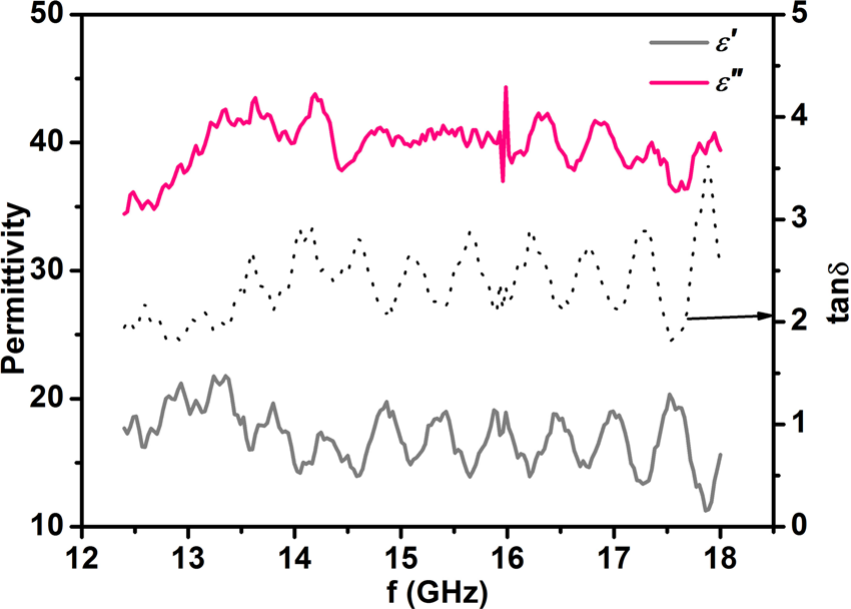
The frequency dependent permittivity (real part *ε*′ and imaginary part *ε*″) and loss tangent (tanδ = *ε*″/*ε*′) of TAC-D ceramic measured at room-temperature. The Ti_3_AlC_2_ ceramics show high imaginary permittivity ε″ and high dielectric loss at the whole Ku band.

Similar to most EMI shielding materials with two-dimensional nano-layered structures, the high SE_A_ of nano-layered Ti_3_AlC_2_ ceramics arises mainly from two major attenuation mechanisms: the high electrical/dielectric loss and layered architecture^1,5,19,20,22^. Figure 4 presents the real (${\varepsilon }^{{\rm{^{\prime} }}}$) and imaginary (ε″) parts of permittivity as well as the dielectric loss tan defined as^9^

6 $$tan\delta ={\varepsilon }^{{\rm{^{\prime} }}{\rm{^{\prime} }}}/{\varepsilon }^{{\rm{^{\prime} }}}$$

of TAC-D ceramic measured at room-temperature. It can be seen that Ti_3_AlC_2_ ceramics exhibit moderate real permittivity around 15 while significantly higher imaginary part of ~40. Consequently, the dielectric loss of Ti_3_AlC_2_ ceramics reaches a considerably high value around 2.5. The high electrical/dielectric loss of Ti_3_AlC_2_ ceramics arises from the abundant free electrons and a large quantity of unpaired defects^16^. The conduction current owing to the skin effect as well as the eddy current will contribute to the ohmic loss, and the unpaired defects will contribute to the dielectric loss. Compared to the electrical/dielectric loss, the multiple reflections also played a significant role in achieving high SE_A_ in nano-layered structures such as graphene and MAX phases, and the multiple reflections are more likely to display microstructure dependences in ceramics. Ti_3_AlC_2_ ceramics exhibit layered grains and therefore abundant flat grain boundaries. When the incident EMI radiations encounter those flat grain boundaries, a portion of the EMI radiations gets immediately reflected due to the impedance mismatch of adjacent layers with the others penetrating through these interfaces. The penetrated EMI radiations can be reflected back and forth between the adjacent grain boundaries. These multiple reflections could increase the length of EMI transmission pathway by a large scale and enhance the attenuation of EMI accordingly^1,7,8,20^. In coarse grains with high aspect ratios, a higher degree of multiple reflections can be expected due to the large surface area of a single grain boundary and thereby a higher absorption loss. Consequently, the origin of microstructure dependent EMI shielding effectiveness can be ascribed to the enhanced degree of multiple reflections with increasing level dimensions of layered Ti_3_AlC_2_ grains.

**Figure 5:**
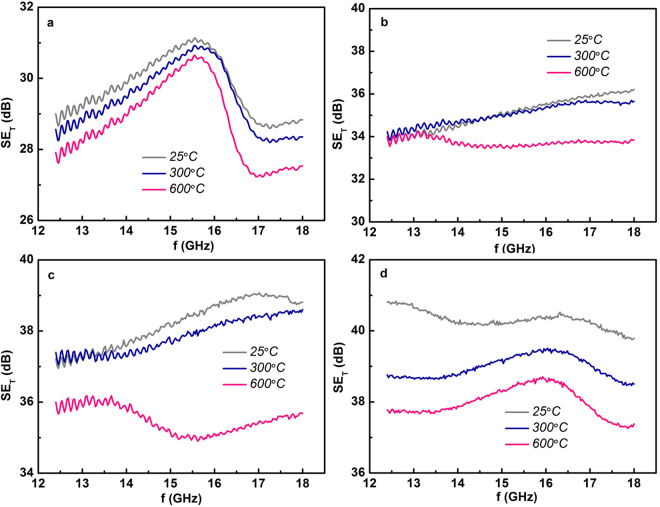
The frequency dependent SE_T_ of various Ti_3_AlC_2_ ceramics measured at different temperatures. (**a**). TAC-A; (**b**). TAC-B; (**c**). TAC-C; (**d**). TAC-D. The shielding effectiveness of all Ti_3_AlC_2_ ceramics exhibit satisfactory temperature stability and high EMI SE still persist at high temperatures.

Figure 5 shows the frequency dependences of SE_T_ at different temperatures up to 600 °C for different Ti_3_AlC_2_ ceramics. It can be seen that the SE_T_ of all Ti_3_AlC_2_ ceramics only shows a slight decrease with increasing temperature. The variations of SE_T_ from room-temperature to 600 °C for all Ti_3_AlC_2_ ceramics is less than 10%. A high SE_T_ around 38 dB was still maintained at 600 °C for TAC-D ceramic, demonstrating that the EMI shielding properties of Ti_3_AlC_2_ ceramics have excellent thermo-stability and is well suitable for high-temperature EMI shielding applications. Figure S4 shows the frequency dependences of SE_R_ and SE_A_ of different Ti_3_AlC_2_ ceramics measured at 600 °C. By comparing Fig. 2 and Fig. S4, it can be easily observed that for all Ti_3_AlC_2_ ceramics the SE_R_ kept almost the same with increasing temperature to 600 °C. Although the electrical conductivity of Ti_3_AlC_2_ ceramics tend to decrease with increasing temperature, the giant impedance mismatch between Ti_3_AlC_2_ ceramics and the air made the SE_A_ almost unaffected by the slight decrease of electrical conductivity. The decrease of SE_T_ solely arises from the decrease of SE_A_ with increasing temperature. The SE_A_ decrease equally for all Ti_3_AlC_2_ ceramics and TAC-D ceramic still exhibits the highest SE_A_ around 26 dB in the Ku-band frequency range at 600 °C. The slight decrease of absorption loss with increasing temperature can be ascribed to the less internal friction of dipole reorientation at high temperatures.

## Conclusions

Various Ti_3_AlC_2_ ceramics with distinct microstructures were prepared by pressureless sintering and hot-pressing. Their EMI shielding properties were characterized at Ku-band frequency range from room temperature to 600 °C. It was found that the EMI shielding properties of Ti_3_AlC_2_ ceramics display remarkable microstructure dependences. High EMI shielding effectiveness over 40 dB was obtained in coarse-grained Ti_3_AlC_2_ ceramic and the excellent shielding properties were well maintained at high temperatures up to 600 °C. The variations of EMI SE_T_ with changing microstructure dominantly arises from the variation of absorption loss SE_A_ which is related to the high electric/dielectric loss and more importantly the nano-layered structure of Ti_3_AlC_2_. Coarse Ti_3_AlC_2_ grains with high aspect ratio are more favourable for high multiple reflections of EMI waves and consequently more absorption loss. Highly conductive TiC ceramics without nano-layered structure exhibit almost microstructure-independent shielding properties and much lower absorption loss than that of Ti_3_AlC_2_ ceramics. These results indicate that Ti_3_AlC_2_ ceramic are promising high-temperature EMI shielding materials.

## Methods

Ti_3_AlC_2_ powder (purity ≥ 98%, ∼300 mesh) was purchased from Forsman (Beijing) Scientific Co., Ltd. Four types of Ti_3_AlC_2_ ceramics referred as TAC-A, TAC-B, TAC-C and TAC-D respectively were prepared by different sintering techniques at different temperatures. In the case of TAC-A, the Ti_3_AlC_2_ powder was first cold-pressed into pellets of 50 mm in diameter and 2 mm in thickness under a uniaxial pressure of 50 MPa, and then pressureless-sintered in Ar atmosphere at 1450 °C for 120 min; In the case of TAC-B, TAC-C and TAC-D, the Ti_3_AlC_2_ powder was placed in a 50 mm diameter graphite die and hot-pressed in Ar atmosphere under different conditions: TAC-B was hot-pressed at 1350 °C for 15 min; TAC-C was hot-pressed at 1400 °C for 120 min; TAC-D was hot-pressed at 1400 °C for 120 min followed by a pressureless sintering in Ar atmosphere at 1450 °C for 500 min. During the hot-press sintering a uniaxial pressure of 30 MPa was applied. Two distinct types of TiC ceramics with 50 mm in diameter and 2 mm in thickness were prepared by hot-pressing at 1800 °C for 15 min (TiC-A) and 1950 °C for 2 h (TiC-B) respectively using commercial TiC powders (∼100 nm, Forsman (Beijing) Scientific Co., Ltd.). A uniaxial pressure of 60 MPa was applied during sintering. The bulk density of the prepared ceramics was measured using the Archimedes’ method. Crystalline phases were characterized by X-ray diffraction (XRD). For microstructure characterization, the Ti_3_AlC_2_ samples were polished using SiC papers and diamond suspensions down to 0.5 µm. The well-polished surfaces were further etched in an acid solution with a volume ratio of HF: HNO_3_:H_2_O = 1:1:3 for 45 s. The morphology of different specimens was observed by field emission scanning electron microscopy (FE-SEM). The grain size distribution of Ti_3_AlC_2_ ceramics was analysed on SEM images (with a magnification of 1000×) of polished and etched surfaces using ImageJ software. In order to ensure the accuracy of measurement, at least 1000 grains from the polished and etched surfaces were considered to statistically obtain the grain level dimension distributions.

For EMI SE characterization, specimens with dimensions of 22.86 mm × 10.16 mm × 1.00 mm were cut and polished. The magnitudes of complex scattering parameters (S-parameters) that correspond to reflection (*S*_11_ or *S*_22_) and transmission (*S*_21_ or *S*_12_) in the frequency range of 12.4~18 GHz (Ku band) were determined through wave-guide method using a vector network analyzer (Agilent N5230A). For accuracy of measurement, the device is carefully calibrated with Through-Reflect-Line (TRL) approach. The high-temperature measurement of SE was performed in a waveguide heated by an inner heater at a rate of 10 °C/min. The temperature range is 25–600 °C and each temperature spot was stabilized for 10 min in order to ensure the accuracy of measurement.

## Electronic supplementary material

Supplementary Information
