# Supplementary material for: Dependences of microstructure on electromagnetic interference shielding properties of nano-layered Ti3AlC2 ceramics
Source: Sci Rep. 2018 May 21;8:7935. doi: 10.1038/s41598-018-26256-0 (PMC5962584; doi:10.1038/s41598-018-26256-0)

**Dependences of microstructure on electromagnetic interference shielding properties of nano-layered Ti_3_AlC_2_ ceramics**

**Yongqiang Tan, Heng Luo, Xiaosong Zhou, Shuming Peng,* Haibin Zhang***

Innovation Research Team for Advanced Ceramics, Institute of Nuclear Physics and Chemistry, China Academy of Engineering Physics, Mianyang, 621900, China

* Corresponding authors: pengshuming@caep.cn, [hbzhang@caep.cn](mailto:hbzhang@caep.cn)

**Figure S1**. The XRD patterns of various Ti_3_AlC_2_ ceramics prepared under different sintering conditions. All Ti_3_AlC_2_ ceramics exhibit the same single phase crystal structure without detectable secondary phases.


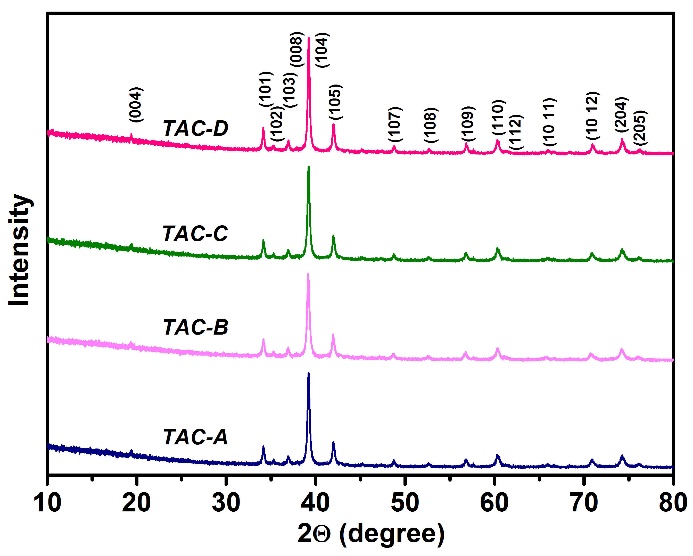


**Figure S2**. Grain size distributions of (a) TAC-A, (b) TAC-B, (c) TAC-C and (d) TAC-D ceramics.


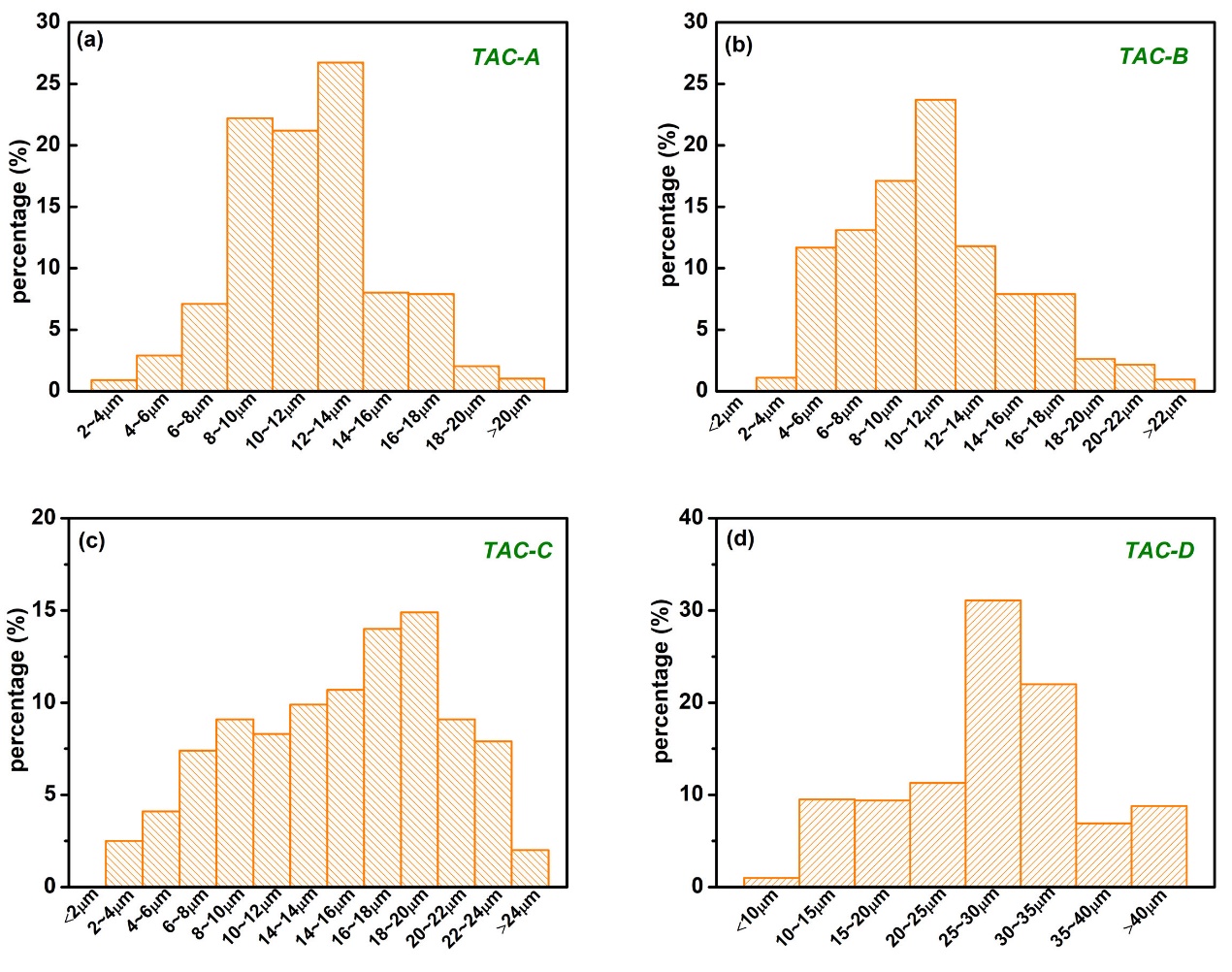


**Figure S3**. Fracture surfaces of TiC ceramics sintered at different temperatures. (a) 1800°C (TiC-A); (b) 1950°C (TiC-B). Both ceramics exhibit dense microstructures and the grain size increases significantly with increasing sintering temperature.


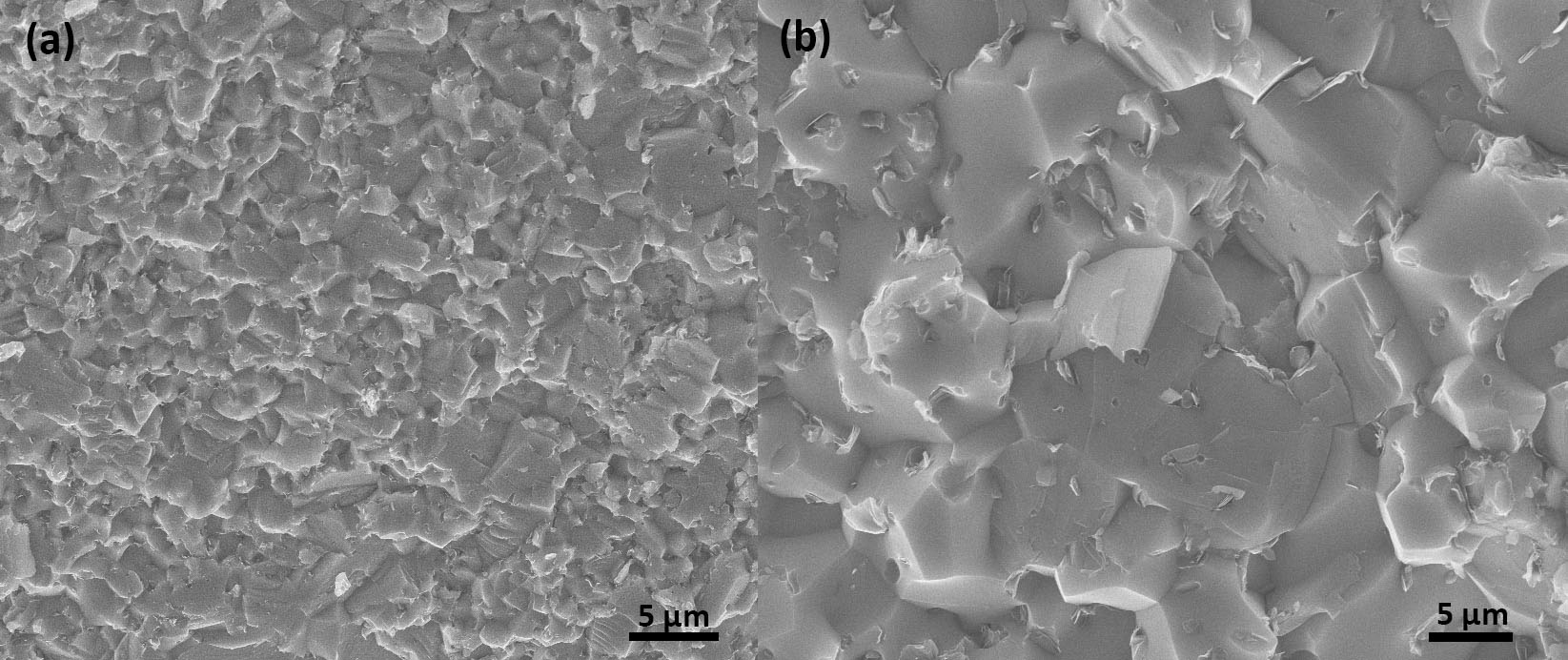


**Figure S4**. The frequency dependences of a. SE_R_ and b. SE_A_ of various Ti_3_AlC_2_ ceramics measured at 600°C. Both SE_R_ and SE_A_ measured at 600°C show similar grain size dependences as measured at room temperature.


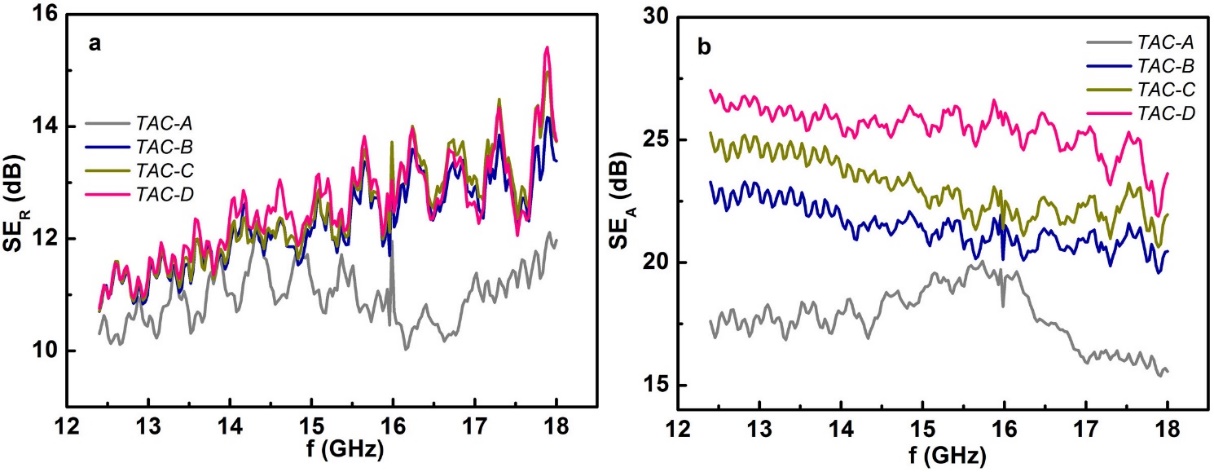

Supplement: Supplementary file 1 — Supplementary Information [file 41598_2018_26256_MOESM1_ESM.docx]
